# Prevalence of Spotted Fever Group *Rickettsia* and *Candidatus* Lariskella in Multiple Tick Species from Guizhou Province, China

**DOI:** 10.3390/biom12111701

**Published:** 2022-11-17

**Authors:** Miao Lu, Chao Meng, Bing Zhang, Xiao Wang, Junhua Tian, Guangpeng Tang, Wen Wang, Na Li, Mengyao Li, Xiaoyu Xu, Yue Sun, Chengyu Duan, Xincheng Qin, Kun Li

**Affiliations:** 1 National Institute for Communicable Disease Control and Prevention, Chinese Center for Disease Control and Prevention, Changping District, Beijing 102206, China; 2 College of Life Sciences, Shandong First Medical University & Shandong Academy of Medical Sciences, Tai’an 271016, China; 3 School of Basic Medical Sciences, Xinjiang Medical University, Urumqi 830011, China; 4 The Military General Hospital of Xinjiang PLA, Urumqi 830000, China; 5 Wuhan Center for Disease Control and Prevention, Wuhan 430024, China; 6 Guizhou Center for Disease Control and Prevention, Guiyang 550004, China; 7 Tianjin Key Laboratory of Food and Biotechnology, Tianjin University of Commerce, Beichen District, Tianjin 300134, China

**Keywords:** *Candidatus* Lariskella guizhouensis, *Rickettsia monacensis*, *Candidatus* Rickettsia jingxinensis, Guizhou Province

## Abstract

Rickettsiales (*Rickettsia* spp., *Ehrlichia* spp., and *Anaplasma* spp., etc.) are generally recognized as potentially emerging tick-borne pathogens. However, some bacteria and areas in China remain uninvestigated. In this study, we collected 113 ticks from mammals in Guizhou Province, Southwest China, and screened for the Rickettsiales bacteria. Subsequently, two spotted fever group *Rickettsia* species and one *Candidatus* Lariskella sp. were detected and characterized. “*Candidatus* Rickettsia jingxinensis” was detected in *Rhipicephalus microplus* (1/1), *Haemaphysalis flava* (1/3, 33.33%), *Haemaphysalis kitaokai* (1/3), and *Ixodes sinensis* (4/101, 3.96%), whereas *Rickettsia monacensis* was positive in *H. flava* (1/3), *H. kitaokai* (2/3), and *I. sinensis* ticks (74/101, 73.27%). At least two variants/sub-genotypes were identified in the *R. monacensis* isolates, and the strikingly high prevalence of *R. monacensis* may suggest a risk of human infection. Unexpectedly, a *Candidatus* Lariskella sp. belonging to the family *Candidatus* Midichloriaceae was detected from *Ixodes ovatus* (1/4) and *I. sinensis* (10/101, 9.90%). The *gltA* and *groEL* gene sequences were successfully obtained, and they show the highest (74.63–74.89% and 73.31%) similarities to “*Candidatus* Midichloria mitochondrii”, respectively. Herein, we name the species “*Candidatus* Lariskella guizhouensis”. These may be the first recovered *gltA* and *groEL* sequences of the genus *Candidatus* Lariskella.

## 1. Introduction

Ticks are one of the most important vectors for pathogens of both humans and animals worldwide [1]. Over the past few decades, more than one hundred tick-borne pathogens, such as spotted fever group Rickettsia, tick-borne encephalitis virus (TBEV), and severe fever with thrombocytopenia syndrome virus (SFTSV), have been identified. In recent years, novel tick-borne pathogens have continued to be reported [2,3]. In 2015, a novel *Anaplasma* species named *Anaplasma capra* was detected in 28 patients with symptoms of fever, headache, and malaise in Northeast China [4]. In 2021, a new tick-borne orthonairovirus named Songling virus (SGLV) was identified in patients from Heilongjiang Province, resulting in the infection of 42 hospitalized patients, with headache, fever, fatigue, and dizziness as the main clinical manifestations [5]. In 2021, Kodama et al. reported a novel tick-borne orthonairovirus named Yezo virus (YEZV) in Japan, associated with acute febrile, thrombocytopenia, and leukopenia [6]. As recently as in 2022, serosurveillance in Japan indicated that a novel tick-borne thogotovirus named Oz virus may naturally infect humans and some other mammals [7]. With increased sampling and development in detection techniques, more novel tick-borne human pathogens are still being discovered.

Due to the vast territory and different climates across China, ticks and tick-borne diseases are prevalent in most areas of this country and pose a significant threat to public health [8]. At least 103 tick-borne agents have been detected, 65 of which were identified in the past two decades [8]. However, most studies have focused on common and easily available tick species, such as *Rhipicephalus microplus*, *Haemaphysalis longicornis*, and *Ixodes persulcatus*. Most of the currently known tick-borne agents were reported in these common tick species [8]. Hence, the pathogens harboured by many tick species may have long been underestimated and largely unexplored due to biased sampling.

The order Rickettsiales includes a group of well-recognized human pathogens such as *Rickettsia* spp., *Orientia* spp., *Ehrlichia* spp., *Anaplasma* spp. and *Neoehrlichia* spp., most of which are tick-borne pathogens [9]. As notorious human pathogens causing a series of symptoms from fever to even death, *Rickettsia*, *Orientia*, *Ehrlichia*, *Neoehrlichia*, and *Anaplasma,* have drawn attention worldwide and been well-studied in many countries and regions [8,10,11]. With the development of high throughput sequencing, some novel families and genera within the order Rickettsiales have been characterized and defined. In 2006, an endosymbiont of the *Ixodes ricinus* named “*Candidatus* Midichloria mitochondrii” was described, which also represents a novel genus *Candidatus* Midichloria within the order Rickettsiales [12]. In 2013, a novel family named “*Candidatus* Midichloriaceae” was proposed, including genera *Candidatus* Midichloria, *Candidatus* Lariskella, *Candidatus* Nicolleia, *Candidatus* Fokinia, and *Candidatus* Aquarickettsia [13]. Although some have been reported to infect humans and mammals such as dogs and horses [14,15], studies on the prevalence, genetic diversity, and pathogenicity of these bacteria are very rare.

To improve the current knowledge on the distribution of ticks and tick-borne Rickettsiales pathogens, ticks collected from Qiandongnan Miao-and-Dong Autonomous Prefecture of Guizhou Province, Southwest China, were thoroughly investigated.

## 2. Materials and Methods

### 2.1. Sample Collection and DNA Extraction

From November to October 2021, ticks were collected in Cengong County (108.82 °O, 27.18 °N), Qiandongnan Miao-and-Dong Autonomous Prefecture, located in Guizhou Province, Southwest China. All the ticks were removed from the body surface of goats and dogs using tweezers, and then stored in 75% alcohol. Morphological identification, mainly based on the characteristic of the anal groove, was carried out by an experienced acarologist to determine the tick species initially. To confirm these results, molecular identification was performed by PCR amplifying and sequencing the partial mitochondrial cytochrome oxidase I (*COI*) gene of randomly selected tick samples, as described previously [14]. After being washed with Phosphate Buffered Saline (PBS) and thoroughly ground, each tick was subjected to DNA extraction individually using a Mollusc DNA extraction kit (Omega, Norcross, GA, USA) following the instructions. The eluted DNA (60 μL) was stored in a −80 °C refrigerator until molecular identification and PCR detection.

### 2.2. Molecular Detection of Rickettsiales Bacteria

The extracted DNA was screened by PCR analysis of a conserved region of *rrs* gene to confirm that the bacteria belong to the order Rickettsiales. PCR was performed using Sensoquest PCR System LabCycler (Sensoquest, Göttingen, Germany). As previously shown, PCR amplification screening *Rickettsia* generates approximately 900 bp products, whereas PCR detecting Anaplasmataceae bacteria generates approximately 450 bp products [16]. The DNA of *R. japonica* and *A. marginale* were used as positive controls, whereas ddH_2_O was set as the negative control. After electrophoresis, all the PCR products that met the expected size were subjected to DNA sequencing. The recovered *rrs* sequences were then aligned to those in the GenBank Database to preliminarily determine their genus or species.

### 2.3. PCR Amplification, Sequencing, Genetic and Phylogenetic Analysis of Key Genes

The citrate synthase gene (*gltA*, 996 bp), 60 kDa chaperonin gene (*groEL*, 1026–1030 bp), outer membrane protein A gene (*ompA*, 706–718 bp), as well as a longer fragment of *rrs* gene (1214–1215 bp), were successfully amplified from the randomly selected *Rickettsia*-positive samples using primers as shown previously [16,17]. For the detected *Candidatus* Lariskella isolates, primers amplifying the *gltA* (456 bp) and *groEL* (651 bp) genes were designed (Table S1). The primers were based on the nucleotide sequences of other *Candidatus* Midichloriaceae members (“*Candidatus* Midichloria mitochondrii”, “*Candidatus* Fokinia cryptica”, and “*Candidatus* Jidaibacter acanthamoeba”), due to the absence of sequences from the genus *Candidatus* Lariskella.

The obtained sequences were aligned with the reference sequences in the GenBank Database with BLASTn algorithm to determine their identities to the reported strains. To perform the phylogenetic analysis, the nucleotide sequences of *Rickettsia* isolates were first manually aligned with those downloaded from the GenBank using the ClustalW method in the MEGA software v3.0. For the *Candidatus* Lariskella isolates, the *rrs* sequences were aligned with other *Candidatus* Lariskella isolates and some *Candidatus* Midichloriaceae members, whereas the *gltA* and *groEL* sequences were only aligned with other *Candidatus* Midichloriaceae members and some Rickettsiaeae members due to the unavailability of *Candidatus* Lariskella sequences in GenBank. Phylogenetic trees were constructed based on the maximum likelihood (ML) method by PhyML v3.2 in the GTR+I+G model [18]. The confidence values for each branch were determined by bootstrap analysis with 100 repetitions. All the trees were mid-point rooted and only bootstrap values >70% are shown.

All the sequences in this study have been uploaded to the GenBank Database (shown in Table S2).

## 3. Results

### 3.1. Sample Collection and Tick Identification

**Figure 1:**
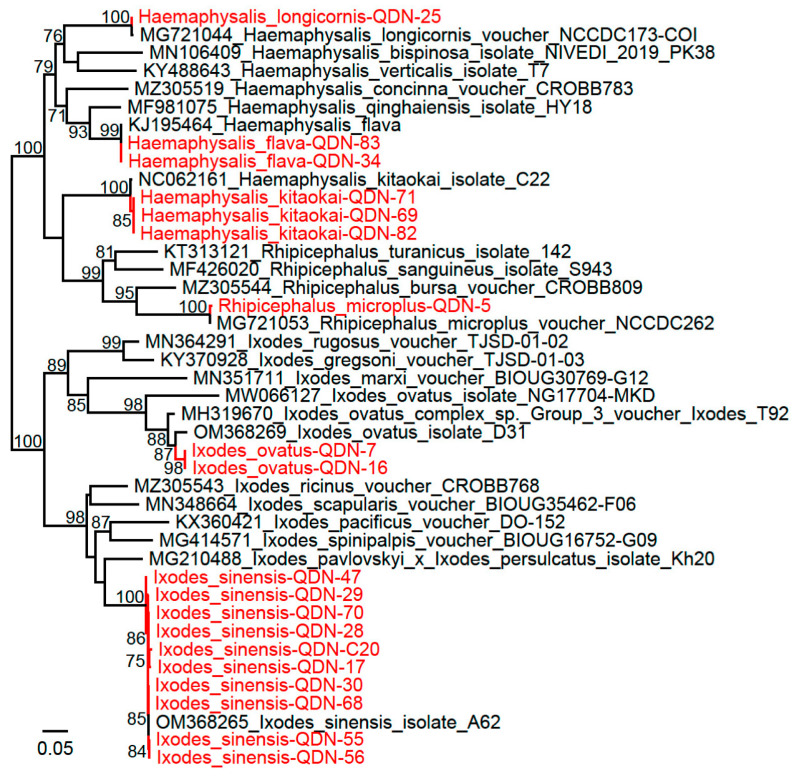
Phylogenetic trees based on the *COI* sequences of ticks from Guizhou Province. Red: Sequences obtained in this study.

From November to October 2021, a total of 113 ticks were collected (112 from goats and 1 from a dog) in Cengong County, Qiandongnan Prefecture. Morphological and molecular identification confirmed the existence of 6 tick species: 1 *Rhipicephalus microplus*, 1 *Haemaphysalis longicornis*, 3 *Haemaphysalis flava*, 3 *Haemaphysalis kitaokai*, 4 *Ixodes ovatus*, and 101 *Ixodes sinensis*. BLASTn shows that all the obtained *COI* sequences (GenBank Accession Numbers: OP107272-OP107273, OP107278-OP107354) have higher than 99% identities to reference *COI* sequences in the GenBank except for the *I. ovatus* ticks which have lower identities of approximately 96%. This is consistent with previous reports that *I. ovatus* contain different phylogenetic groups with remarkable intergroup genetic distances [19]. To our knowledge, this is the first report that *H. flava*, *H. kitaokai*, *I. ovatus*, and *I. sinensis* are present in Guizhou Province. Phylogenetic analysis results are consistent with the BLASTn results (Figure 1), indicating the remarkable diversity of tick species in this area.

### 3.2. Detection and Characterization of Rickettsia Isolates

Based on DNA sequencing and sequence analysis, a total of two *Rickettsia* species were initially determined: *Rickettsia monacensis* and “*Candidatus* Rickettsia jingxinensis”. “*Candidatus* Rickettsia jingxinensis” was detected in seven tick samples: *R. microplus* (1/1, 100%), *H. flava* (1/3, 33.33%), *H. kitaokai* (1/3, 33.33%), and *I. sinensis* (4/101, 3.96%) (Table 1). The *rrs* (1214 bp), *gltA* (996 bp), *groEL* (1026 bp), and *ompA* (706 bp) gene sequences of the two isolates were all identical to the reported “*Candidatus* Rickettsia jingxinensis” isolates from elsewhere in China.

**Figure 2:**
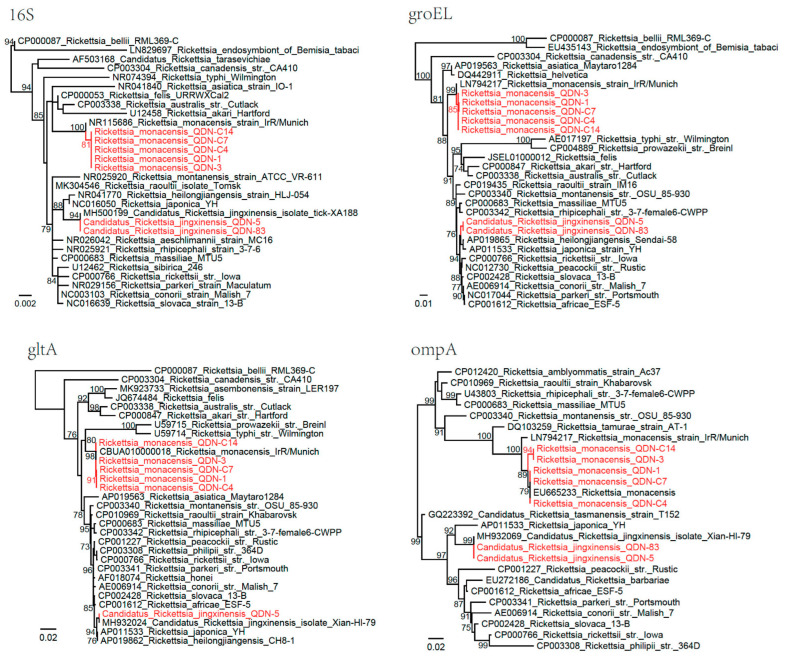
Phylogenetic trees based on the nucleotide sequences of 16S rRNA (*rrs*), *groEL*, *gltA*, and *ompA* genes of *Rickettsia* isolates. Red: Sequences obtained in this study.

A total of 77 ticks were positive for *R. monacensis* including *H. flava* (1/3, 33.33%), *H. kitaokai* (2/3, 66.67%), and *I. sinensis* (74/101, 73.27%) (Table 1). The *rrs* gene (1215 bp) sequences were identical to strains (KX987304-KX987306) previously identified in *I. sinensis* from Wuhan City, Hubei Province, China. The *groEL* sequences (1030 bp) have 99.61–99.71% identity to *R. monacensis* strain IrR/Munich (LN794217). Interestingly, for the *gltA* (996 bp) and *ompA* (712–718 bp) genes, sequences from QDN-1, QDN-C4, and QDN-C7 isolates were identical, whereas those from QDN-3 and QDN-C14 were identical. Compared to the *R. monacensis* strain IrR/Munich, the *ompA* sequences of all five isolates have a six-nucleotide insertion (QDN-1, QDN-C4, and QDN-C7 isolates: ATATAT; QDN-3 and QDN-C14 isolates: AAATAT), whereas QDN-1, QDN-C4, and QDN-C7 isolates have an additional six-nucleotide insertion (CTATAG). In the phylogenetic tree based on the *ompA* gene, isolates were divided into two clusters (Figure 2) which have only 96.62–97.05% homology to the strain IrR/Munich, but were identical to *R. monacensis* isolates from Henan (EU665233) and Anhui (EU665232) Provinces, respectively (both with 81% coverage).

### 3.3. Detection and Characterization of Candidatus Lariskella Isolates

Electrophoresis indicated that 11 tick samples were positive for the Anaplasmataceae bacteria. Unexpectedly, sequencing results showed that all of them belong to the genus *Candidatus* Lariskella, the family *Candidatus* Midichloriaceae. Of the 11 isolates, 1 was from *I. ovatus* (1/4, 25.00%) and 10 were from *I. sinensis* (10/101, 9.90%) (Table 1). The amplified *rrs* sequences (468 bp) have the highest (99.36–99.57%) identity to *Candidatus* Lariskella sp. isolates identified in *I. sinensis* from Wuhan City (KX987316, KX987317) and 98.72–98.93% to the “*Candidatus* Lariskella arthropodarum” clone AmLaKka1 (JQ726736) detected in *Arocatus melanostomus* from Japan.

**Figure 3:**
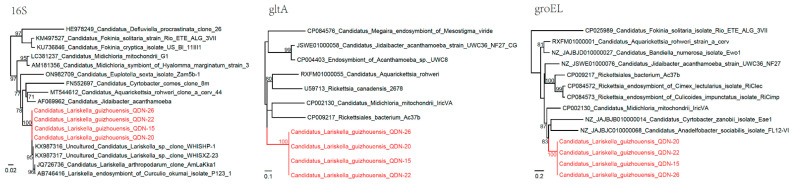
Phylogenetic trees based on the nucleotide sequences of 16S rRNA (*rrs*), *gltA*, and *groEL* genes of *the Candidatus* Lariskella isolates. Red: Sequences obtained in this study.

For further study of the detected *Candidatus* Lariskella isolates, we tentatively designed primers to amplify the *gltA* and *groEL* genes based on sequences of other genera of the family *Candidatus* Midichloriaceae. Subsequently, both the *gltA* (410–453 bp) and *groEL* (651 bp) were successfully recovered from four randomly selected isolates. BLASTn alignment showed that the two gene sequences were mostly related to “*Candidatus* Midichloria mitochondrii”. The *gltA* sequences have 74.63–74.89% identity to “*Candidatus* Midichloria mitochondrii” IricVA (CP002130), and 74.63–74.71% identity to “*Candidatus* Nicolleia massiliensis” (DQ788563), both of which belong to the family *Candidatus* Midichloriaceae, the order Rickettsiales. Meanwhile, the *groEL* sequences showed 73.31% identity to “*Candidatus* Midichloria mitochondrii” IricVA (CP002130), and 71.21% to Rickettsiales bacterium Ac37b (CP009217). To the best of our knowledge, these may be the first obtained *gltA* and *groEL* sequences of the genus *Candidatus* Lariskella. In the phylogenetic tree based on the *gltA* and *groEL* genes, all four isolates were surrounded by other *Candidatus* Midichloriaceae members as well as several Rickettsiaceae members, but they were distinct from any other bacterial species and formed an independent clade (Figure 3). All of these results indicated the representation of an unrecognized species. Herein, we name it “*Candidatus* Lariskella guizhouensis”.

## 4. Discussions

To date, approximately 124 tick species belonging to 11 genera have been recorded in China [8]. Although the geographic distribution of various tick species has been well studied, some neglected areas are still largely unexplored. Herein, we collected 113 ticks in Guizhou Province, Southwest China, and molecularly validated the existence of six tick species in them. Of those, *I. sinensis*, *H. flava*, *H. kitaokai*, and *I. ovatus* had never been recorded in this province. These results may contribute to our knowledge of the geographic distribution of ticks and tick-borne pathogens in China.

Two *Rickettsia* species were detected in tick samples: *Rickettsia monacensis* and “*Candidatus* Rickettsia jingxinensis”. As a widely distributed spotted fever group *Rickettsia*, “*Candidatus* Rickettsia jingxinensis” has been detected in *R. microplus* and *H. longicornis* from multiple provinces in China (Liaoning, Guangxi, Sichuan, Hebei, Shaanxi, and Yunnan) and other Asian countries (Korea and India) [16,17,20,21,22]. Our results have demonstrated the circulation of “*Candidatus* Rickettsia jingxinensis” in Guizhou Province. Due to its wide geographic distribution and host range, the potential risk to the public health of this *Rickettsia* should be evaluated. In this study, *R. monacensis* was detected in three tick species with a total prevalence of 68.14%. Due to the fact that all the ticks were removed from domestic animals, the possibility remains that the detected pathogens are from the blood of infected hosts the ticks fed on. *Rickettsia monacensis* was first identified in *Ixodes ricinus* ticks from Germany [23] and has also been detected in other tick species such as *Ixodes boliviensis*, *I. sinensis*, *Rhipicephalus sanguineus*, *Hyalomma impeltatum* [24,25,26], as well as some mammal hosts such as camels and bats [26,27]. As an SFGR member, *R. monacensis* was recognized as a human pathogen causing Mediterranean Spotted Fever-like rickettsioses [25]. Although *R. monacensis* has been detected in several provinces of China [25,28,29], no human infection cases were reported until now. The strikingly high prevalence of *R. monacensis* in ticks (especially in *I. sinensis*) may indicate the risk of human infection, especially in populations who are frequently in contact with domestic animals. Furthermore, genetic and phylogenetic analysis indicated that two variants/sub-genotypes of the *R. monacensis* isolates may have been generated, indicating its long-term evolution in this area. The pathogenicity of the variants/sub-genotypes may warrant further studies.

First proposed in 2012, the genus *Candidatus* Lariskella has been scarcely studied [30], and little is known about its genetic features. Even with respect to the first identified and most studied species, “*Candidatus* Lariskella arthropodarum” (previously named Montezuma) [14], only some *rrs* sequences were available in the GenBank Database until now. Notably, “*Candidatus* Lariskella arthropodarum” has been detected in acutely febrile patients with the bites of *Ixodes* ticks in the Far East of Russia [14], suggesting its potential role as a tick-borne human pathogen. In this study, we detected a *Candidatus* Lariskella species in *Ixodes* ticks from Guizhou Province, and successfully obtained the *gltA* and *groEL* sequences. To our knowledge, these are the first reported *gltA* and *groEL* sequences of the genus *Candidatus* Lariskella. The remarkable genetic distance between this species and other *Candidatus* Lariskella species indicates that it represents a novel species. Herein we named it “*Candidatus* Lariskella guizhouensis”. It is of interest whether “*Candidatus* Lariskella guizhouensis” can be transmitted to humans and animals through tick bites.

## 5. Conclusions

In conclusion, a high prevalence of *Rickettsia monacensis* was observed in ticks from Guizhou Province, and they represent at least two variants/sub-genotypes. For this reason, the risk of human infection among local people should be evaluated. In addition, a *Candidatus* Lariskella sp. belonging to the family *Candidatus* Midichloriaceae was detected and characterized. To the best of our knowledge, this is the first report of *gltA* and *groEL* sequences of *Candidatus* Lariskella. Genetic and phylogenetic analysis indicates that it represents a novel species: “*Candidatus* Lariskella guizhouensis”. This study may contribute to our knowledge of the extensive genetic diversity of Rickettsiales bacteria, and highlights that their potential threat to human health should be further investigated.

## Figures and Tables

**Table 1:** Prevalence of *Rickettsia* and “*Candidatus* Lariskella guizhouensis” in different tick species from Guizhou Province.

	“*Ca.* Rickettsia Jingxinensis”	*Rickettsia monacensis*	“*Ca.* Lariskella Guizhouensis”
*R. microplus*	1/1 (100%) ^a^	0/1 (0.00%)	0/1 (0.00%)
*H. longicornis*	0/1 (0.00%)	0/1 (0.00%)	0/1 (0.00%)
*H. flava*	1/3 (33.33%)	1/3 (33.33%)	0/1 (0.00%)
*H. kitaokai*	1/3 (33.33%)	2/3 (66.67%)	0/3 (0.00%)
*I. ovatus*	0/4 (0.00%)	0/4 (0.00%)	1/4 (25.00%)
*I. sinensis*	4/101 (3.96%)	74/101 (73.27%)	10/101 (9.90%)
Total	7/113 (6.19%)	77/113 (68.14%)	11/113 (9.73%)

^a^ positive samples/total samples.

## Data Availability

All sequence files are available from the NCBI database (Accession Numbers shown in Table S2).

## Supplementary Materials

The following supporting information can be downloaded at: https://www.mdpi.com/article/10.3390/biom12111701/s1, Table S1. The primers used for amplification of *gltA* and *groEL* genes from *Candidatus* Lariskella guizhouensis by hemi-nested PCR. Table S2. Genbank numbers of the *rrs*, *gltA*, *groEL*, and *ompA* sequences of Rickettsia and “*Candidatus* Lariskella guizhouensis” in this study.
