# Supplementary material for: Prevalence of Spotted Fever Group Rickettsia and Candidatus Lariskella in Multiple Tick Species from Guizhou Province, China
Source: Biomolecules. 2022 Nov 17;12(11):1701. doi: 10.3390/biom12111701 (PMC9688252; doi:10.3390/biom12111701)
Supplement: Supplementary file 1 [file biomolecules-12-01701-s001.zip › Table S1.pdf]

Table S1. The primers used for amplification of *gltA* and *groEL* genes from *Candidatus* Lariskella guizhouensis by hemi-nested PCR.

**Primer**	**Gene**	**Cycle**	**Sequence**	**Anticipated Amplicon Length**
LariEL-ex5	*groEL*	1	5-CCWMAADTYACWAARGATGGWGT-3	450 bp
LariEL-ex3	*groEL*	1	5-TTKNARYTTYTCYTTATCRTADTC-3	
LariEL-in3	*groEL*	2	5-TTTCKTCTRTCDCCRAANCC-3	
LariGltex5	*gltA*	1	5-CATGCTGATCAYGARCAAAATGC-3	650 bp
LariGlt3	*gltA*	1	5-TCTTCHGGAGCYAAYCCDTTTG-3	
LariGltin5	*gltA*	2	5-TCWTCVATCATTTCTTTCCATTGAG-3	
