# Supplementary material for: Prevalence of Spotted Fever Group Rickettsia and Candidatus Lariskella in Multiple Tick Species from Guizhou Province, China
Source: Biomolecules. 2022 Nov 17;12(11):1701. doi: 10.3390/biom12111701 (PMC9688252; doi:10.3390/biom12111701)
Supplement: Supplementary file 1 [file biomolecules-12-01701-s001.zip › Table S2.pdf]

Table S2. Genbank numbers of the *rrs*, *gltA*, *groEL*, and *ompA* sequences of *Rickettsia* and *Candidatus* Lariskella guizhouensis in this study.

No.	**Gene**	**Genbank Number**	**Bacterial Strain**
1	*rrs*	OP115561	*Candidatus*_Lariskella_guizhouensis_QDN-15
2	*rrs*	OP115562	*Candidatus*_Lariskella_guizhouensis_QDN-20
3	*rrs*	OP115563	*Candidatus*_Lariskella_guizhouensis_QDN-22
4	*rrs*	OP115564	*Candidatus*_Lariskella_guizhouensis_QDN-26
5	*gltA*	OP125507	*Candidatus*_Lariskella_guizhouensis_QDN-15
6	*gltA*	OP125508	*Candidatus*_Lariskella_guizhouensis_QDN-20
7	*gltA*	OP125509	*Candidatus*_Lariskella_guizhouensis_QDN-22
8	*gltA*	OP125510	*Candidatus*_Lariskella_guizhouensis_QDN-26
9	*groEL*	OP125511	*Candidatus*_Lariskella_guizhouensis_QDN-15
10	*groEL*	OP125512	*Candidatus*_Lariskella_guizhouensis_QDN-20
11	*groEL*	OP125513	*Candidatus*_Lariskella_guizhouensis_QDN-22
12	*groEL*	OP125514	*Candidatus*_Lariskella_guizhouensis_QDN-26
13	*rrs*	OP107869	*Candidatus*_Rickettsia_jingxinensis_QDN- 5
14	*rrs*	OP107870	*Candidatus*_Rickettsia_jingxinensis_QDN- 83
15	*rrs*	OP107871	*Rickettsia_monacensis*_QDN-1
16	*rrs*	OP107872	*Rickettsia_monacensis*_QDN-3
17	*rrs*	OP107873	*Rickettsia_monacensis*_QDN-C4
18	*rrs*	OP107874	*Rickettsia_monacensis*_QDN-C7

19	*rrs*	OP107875	*Rickettsia_monacensis*_QDN-C14
20	*gltA*	OP125487	*Candidatus*_Rickettsia_jingxinensis_QDN-5
21	*gltA*	OP125488	*Rickettsia_monacensis*_QDN-1
22	*gltA*	OP125489	*Rickettsia_monacensis*_QDN-3
23	*gltA*	OP125490	*Rickettsia_monacensis*_QDN-C4
24	*gltA*	OP125491	*Rickettsia_monacensis*_QDN-C7
25	*gltA*	OP125492	*Rickettsia_monacensis*_QDN-C14
26	*groEL*	OP125493	*Candidatus*_Rickettsia_jingxinensis_QDN-5
27	*groEL*	OP125494	*Candidatus*_Rickettsia_jingxinensis_QDN-83
28	*groEL*	OP125495	*Rickettsia_monacensis*_QDN-1
29	*groEL*	OP125496	*Rickettsia_monacensis*_QDN-3
30	*groEL*	OP125497	*Rickettsia_monacensis*_QDN-C4
31	*groEL*	OP125498	*Rickettsia_monacensis*_QDN-C7
32	*groEL*	OP125499	*Rickettsia_monacensis*_QDN-C14
33	*ompA*	OP125500	*Candidatus*_Rickettsia_jingxinensis_QDN-5
34	*ompA*	OP125501	*Candidatus*_Rickettsia_jingxinensis_QDN-83
35	*ompA*	OP125502	*Rickettsia_monacensis*_QDN-1
36	*ompA*	OP125503	*Rickettsia_monacensis*_QDN-3
37	*ompA*	OP125504	*Rickettsia_monacensis*_QDN-C4
38	*ompA*	OP125505	*Rickettsia_monacensis*_QDN-C7
39	*ompA*	OP125506	*Rickettsia_monacensis*_QDN-C14
